# The Pregnancy Outcomes Among Newly Arrived Asylum-Seekers in Italy: Implications of Public Health

**DOI:** 10.1007/s10903-020-01126-y

**Published:** 2020-12-05

**Authors:** Lucia Fontanelli Sulekova, Martina Spaziante, Serena Vita, Paola Zuccalà, Valentina Mazzocato, Ornella Spagnolello, Maurizio Lopalco, Laura Elena Pacifici, Luca Bello, Cristian Borrazzo, Silvia Angeletti, Massimo Ciccozzi, Giancarlo Ceccarelli

**Affiliations:** 1 Department of Public Health and Infectious Diseases, Policlinico Umberto I Hospital. University of Rome Sapienza, Rome, Italy; 2 Migrant and Global Health Organization (Mi-HeRO), Rome, Italy; 3 Auxilium Soc Coop. Sanitary Bureau of Asylum Seeker Center of Castelnuovo di Porto, Senise, Italy; 4 Italian Red Cross, Sanitary Bureau of) Extraordinary Reception Centers for Migrants “ENEA”, “Pietralata” and “Penelope” of Rome, Rome, Italy; 5 Emergency NGO, Milan, Italy; 6 Emergency Department, Policlinico Umberto I Hospital, University of Rome Sapienza, Rome, Italy; 7 UniCamillus – Saint Camillus International University of Health Sciences, Rome, Italy; 8 Unit of Gynecology, Department of Obstetrics and Gynecology, University of Turin, Maria Vittoria Hospital, Turin, Italy; 9 Statistical Unit, Department of Public Health and Infectious Diseases, University of Rome Sapienza, Rome, Italy; 10 Unit of Clinical Laboratory Science, University Campus Bio-Medico of Rome, Rome, Italy; 11 Unit of Medical Statistics and Molecular Epidemiology, University Campus Bio-Medico of Rome, Rome, Italy; 12 Italian Red Cross, Metropolitan Area of Rome Committee, Sanitary Direction of Reception Centers for Migrants, Rome, Italy

**Keywords:** Pregnancy outcome, Induced abortion, Migrants, Asylum seekers, Reproductive health

## Abstract

**Background:**

Migration has a significant impact on overall health and pregnancy outcome. Despite the fact that growing volume of migration flows significantly engaging the public health system of European host countries, there is a lack of evidence concerning pregnancy outcomes of newly arrived asylum-seeking women.

**Methods:**

Data about pregnant asylum seekers hosted in the Italian Reception Centers between the 1 st June 2016 and the 1st June 2018 were retrospectively collected and analysed in the present study. We examined the following pregnancy outcomes: miscarriage, self-induced abortion, voluntary pregnancy termination, live-birth; and studied potentially related socio-demographic factors.

**Results:**

Out of the 110 pregnant women living in the reception centers, 44 (40%) had eutocic delivery, 8 (7.3%) dystocic delivery, 15 (13.6%) miscarriage, 17 (15.5%) self-induced abortion and 26 (23.6%) underwent voluntary pregnancy termination. Nigerian women were at a significantly higher risk of abortive outcomes for voluntary pregnancy termination (*p* < 0.001), miscarriage (*p* = 0.049) and self-induced abortion (*p* < 0.001). Being unmarried was significantly associated with voluntary pregnancy termination and self-induced abortion. Women who chose to undergo unsafe abortion did not result to have significantly lower educational levels, compared to women who preferred medical abortion.

**Conclusion:**

This study offers first insights into pregnancy outcomes among asylum-seeking women in Italy. The country of origin and marital status seem to significantly impact on pregnancy outcome. We identified sub-groups of migrant women at increased risk of abortive outcomes, and highlight the need to improve care in order to promote migrant women’s reproductive health.

## Introduction

In 2015 and 2016, a large increase in arrivals of migrants and refugees fleeing protracted conflict, poverty, and persecution seeking security and economic opportunities was recorded in Europe. In 2016, out of the total migrants arriving in Italy, the largest number came from Nigeria – 37,551 more than 20% of total arrivals, Eritreans were the second largest group at 20,718 (11%), followed by Côte d’Ivoire, Guinea, Gambia (7% each), Senegal, Mali (6% each), Sudan, (5%), Bangladesh and Somalia (4% each) [1].

About a quarter of all refugees and internally displaced persons worldwide are women, which represent a highly vulnerable group in terms of sexual and reproductive health. Migration has a significant impact on their overall health, sexual behaviour, use of contraception, pregnancy outcomes [2] and their perceived attitude to induced abortion [3]. Refugee women are at a high risk of sexual victimisation, often are forced to pay for their migration through prostitution or are subject to sexual assault during their journey or during their staying in Libyan prisons [4]. According to Trovato et al., 11% of migrant women who arrived in Italy were pregnant [5], 30.2% became pregnant after sexual abuse [6]. The risk for sexual abuse is higher among women who travelled alone compared to those who travelled with partners, family members or friends [7].

Regular or irregular migrants within the territory of the Italian state have access to health coverage through a specific system called “Temporarily Present Foreigners” (*Stranieri Temporaneamente Presenti* – STP), a short-term but renewable anonymous code that migrants can request at their Local Healthcare Provider. STP ensures access to free basic health care coverage. Indeed, urgent and essential health treatments are guaranteed to migrants all over the national territory. Abortion is performed free-of-charge at both public health care facilities part of the National Health Care System and at private facilities contracted and authorized by regional health authorities. According to the Italian abortion low (*legge 104/1978),* in Italy one is allowed to have an abortion within 90 days of gestation; after 90 days it is possible only in the case of severe reasons. Migrants face multiple issues regarding healthcare services in their host country. The access to perinatal health care system is affected by administrative problems, lack of information, fear of authorities, language barriers, low level of health literacy and cultural differences, those factors have an impact even on pregnancy outcomes [8].

Pregnant refugees have higher rates of adverse pregnancy outcomes, including caesarean section [9], abortive outcomes/stillbirth [10, 11], and other maternal and perinatal morbidities [12]. In many European countries, migrant women have higher voluntary pregnancy termination (VPT) rates than the native population [13]. Another problem concerns repeated VPTs and the recorded increase in the practice of self-induced abortion, often by taking ad hoc drugs purchased through illegal networks [14]. In many cases, these situations are presented as spontaneous abortions, which confirms the fact that the miscarriage rates (4.8‰) have remained substantially unchanged over time among Italian women, while immigrants miscarriage rates resulted variably higher (10.4‰–15.85‰) [15].

Our interest on the phenomenon of VPT and induced abortions among migrant women was raised from the observation that data regarding asylum seekers in industrialized countries are sparse, despite having a major impact on public health.

Therefore, our study aimed to describe the trends in pregnancy outcomes and study potentially related socio-demographic factors.

## Methods

This is a retrospective observational study evaluating as primary endpoint pregnancy outcomes among newly arrived women hosted in ASC (Asylum Seeker Center) and ERCs (Extraordinary Reception Centers for Migrants) in Italy. Miscarriage (spontaneous abortion <22 weeks), voluntary termination of pregnancy (VTP) (legally induced abortion before 12 weeks), reported self-induced abortion and live-birth (eutocic or dystocic delivery), were evaluated as possible outcomes.

### Study Population

The medical records of all female asylum seekers in reproductive age (15–49 years) hosted in five large Reception Centers located in Central Italy between the 1st June 2016 and the 31st December 2018 were retrospectively reviewed. The Centers involved were the Asylum Seeker Center (ASC) of Castelnuovo di Porto (Province of Rome, Italy), the Extraordinary Reception Center for Migrants (ERC) “Mondo Migliore” of Rocca di Papa (Province of Rome, Italy), the ERC “Penelope” of Rome (Italy), the ERC “Pietralata” of Rome (Italy), and the ERC “ENEA” of Rome (Italy).

### Sources of Data

Socio-demographic and medical data on pregnancy outcomes of all newly arrived migrants who were pregnant on arrival or got pregnant during their staying at the Centres were collected.

Clinical data reviewed were previously recorded at the medical ambulatories of ASC and ERCs managed by consultant physicians in internal medicine, infectious diseases, general surgery and gynaecology. The team also included psychologists and nurses. As a standard of care, a complete screening visit was performed upon arrival, as previously described [16–19].

Blood tests were offered free of charge on top of the screening visit and were performed on a voluntary basis following signed informed consent. A rapid urine pregnancy test was available at the ASC and ERCs medical ambulatories upon request at any time when the amenorrhea was present for more than 10 days. Furthermore, the test was offered actively when symptoms compatible with pregnancy were present or in case drugs potentially contraindicated in pregnancy were given. According to data from ERCs and ASC pharmacies, 15 boxes containing 100 rapid pregnancy tests were used during the study period.

Data obtained were confirmed by quantifying the serum human chorionic gonadotropin (hCG). In the event of a positive test, patients were taken in charge by the gynaecological clinic of the nearest hospital where patients were given regular clinical and laboratory monitoring as defined by the guidelines of pregnancy management.

Pregnancy outcomes, including miscarriage, VTP, referred self-induced abortion and live-birth with eutocic or dystocic delivery, were recorded. Indicators of outcome delivery were reported in accordance with the indications of the 2010 European Perinatal Health Report (Euro-PERISTAT) [20, 21]. The study was carried out in accordance with the Helsinki Declaration and the data were collected and analyzed after receiving informed consent. Ethical approval was not required because the study was based on a retrospective analysis of data routinely collected and stored according to the Italian law on privacy (General authorization to process personal data for scientific research purposes granted by the Italian Data Protection Authority (1 March 2012 as published in Italy’s Official Journal no. 72 dated 26 March 2012)).

### Statistical Analyses

All data were analyzed using Statistical Package for Social Science (SPSS) version 20 or Microsoft Excel (Office 2018). Description of mean ± standard deviation (±SD), range (minimum – maximum), proportions (or percentages) and rates of the given data on each variable has been calculated. The demographic characteristics of patients were compared using t-test for continuous variable and chi-square test for categorical variables. A *p* value of less than 0.05 was considered statistically significant.

## Results

### Characteristics of the Cohort

**Fig. 1:**
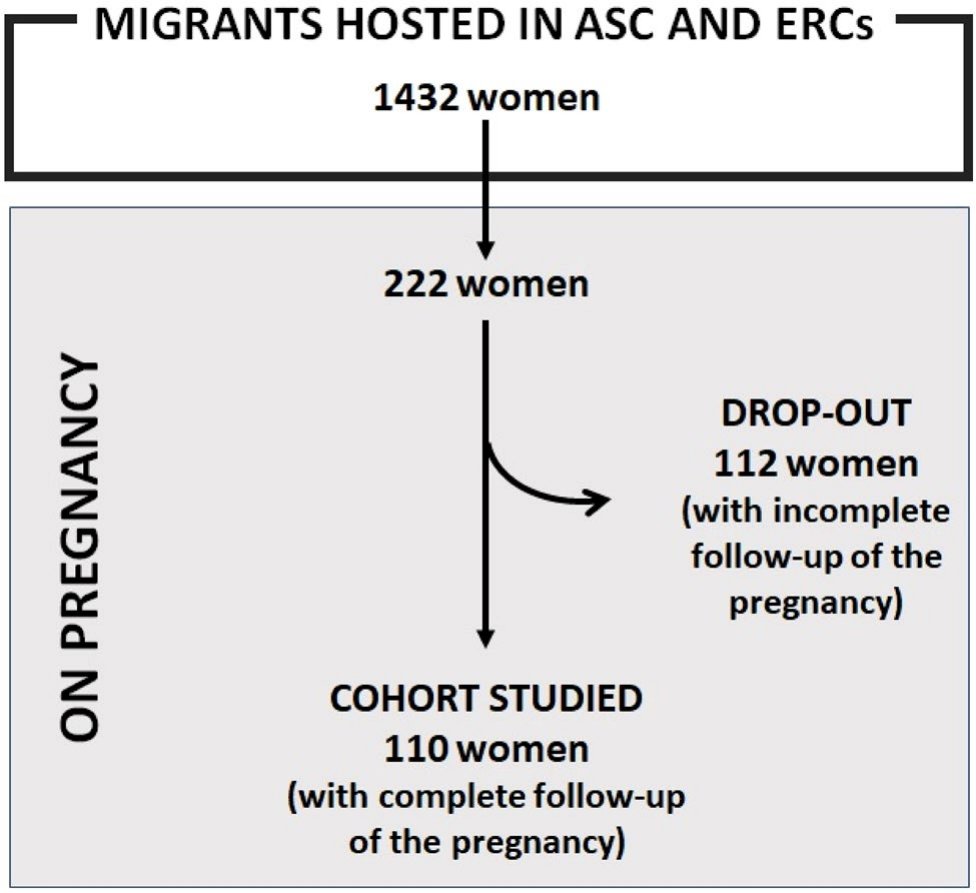
Enrolment of the cohort

Out of 1432 migrant women hosted at the ERCs and ASC during the study period, 222 (15.5%) were pregnant on arrival or got pregnant during their stay in the centers. 110 women had a complete follow-up of pregnancy and were enrolled in the study; on the other hand, 112 (50.5%) women were lost to follow-up because they either left voluntarily the centers (47; 42%) or were transferred to another facility (65; 58%) before the pregnancy outcome was known (Fig. 1).

The age of the study population ranged between 15 and 42 years with a mean age of 24 years. Concerning the country of origin, most of the enrolled pregnant women came from Nigeria (61; 55.5%) or Eritrea (31; 28.2%). The remaining 18 women originated from Somalia (4) Côte d’Ivoire (3), Pakistan (2), Syria (2), Egypt (1), Ghana (1), Mali (1), Morocco (1), Senegal (1), Congo (1), and Turkey (1).

### Pregnancy Outcomes

Out of 110 women with complete pregnancy follow-up, 44 (40%) had eutocic delivery, 8 (7.3%) dystocic delivery, 15 (13.6%) miscarriage, 17 (15.5%) reported self-induced abortion and 26 (23.6%) underwent VPT (Table 1).

**Table 1:** Demographic variables and different pregnancy outcomes

Variables (n; %)	Delivery (52; 47.3%)	p value	Miscarriage (15; 13.6%)	p value	Self-induced abortion (17; 15.5%)	p value	VPT (26; 23.6%)	p value
Age; years (mean, SD)	26.2 ± 6.3	–	23.8 ± 5.8	–	24.1 ± 2.1	–	24.3 ± 5.5	–
Education; years (mean, SD)	7 ± 4.6	–	6 ± 5.1	–	6 ± 5.6	–	8 ± 3.4	–
Unmarried status; n (%)	15 (28.8%)	<0.001	10 (66.6%)	0.072	13 (76.5%)	<0.001	17 (65.4%)	0.027
Previous pregnancy; n (%)	33 (63.5%)	0.006	5 (33.3%)	0.067	3 (17.6%)	<0.001	9 (34.6%)	0.027
Children; n (%)	29 (55.8%)	0.239	7 (46.7%)	0.722	3 (17.6%)	<0.001	5 (19.2%)	<0.001
Nigerian women	19 (36.5%)	0.371	7 (46.7%)	0.049	15 (88.2%)	<0.001	20 (76.9%)	<0.001
Eritrean women	24 (46.2%)	<0.001	2 (13.3%)	0.103	0 (0%)	0.153	5 (19.2%)	0.084
Other Countries of origin	9 (17.3%)	0.028	6 (40.0%)	0.715	2 (11.8%)	<0.002	1 (3.8%)	<0.001

*VPT* voluntary termination of pregnancy

Analysing data from our cohort, the “country of origin” seems to significantly impact on pregnancy outcome. Notably, higher delivery rates were recorded among Eritrean women, compared to women originating from Nigeria (24/31 (77%) vs 19/61 (31%), *p* < 0.001) or from other countries (24/31 (77%) vs 9/18 (50%), *p* = 0.055) (Table 2).

**Table 2:** Country of origin and pregnancy outcomes

Variables (n; %)	Eritrean women (31; 28.2%)	Nigerian women (61; 55.5%)	Other Countries of origin (18; 16.3%)	p value		
				Eritrean vs Nigerian	Eritrean vs Other	Nigerian vs Other
Age; years (mean, SD)	26 ± 7.4	24 ± 16.9	26 ± 20.7	0.231	0.223	0.321
Education; years (mean, SD)	8 ± 4.6	6 ± 5.1	5 ± 4.1	0.046	0.001	0.113
Unmarried status; n (%)	8 (26%)	46 (75%)	5 (28%)	<0.001	0.884	<0.001
Children; n (%)	13 (42%)	14 (23%)	2 (11%)	0.060	0.002	0.118
Pregnancy at the arrival; n (%)	25 (81%)	14 (23%)	11 (61%)	<0.001	0.129	0.007
Delivery; n (%)	24 (77%)	19 (31%)	9 (50%)	<0.001	0.055	0.088
Miscarriage; n (%)	2 (10%)	7 (11%)	6 (33%)	0.883	0.047	0.121
Self-induced abortion; n (%)	0 (0%)	15 (25%)	2 (11%)	0.002	0.062	0.098
VPT; n (%)	5 (16%)	20 (33%)	1 (6%)	0.085	0.309	0.003

*VPT* voluntary termination of pregnancy

Interestingly, 15/17 (88.2%) of women who reported self-induced abortion originated from Nigeria, whereas none of them was Eritrean (88.2% vs 0%, *p* = 0.002) and 2/18 (11%) originated from other Countries (88.2% vs 11%, *p* = 0.062).

The woman’s age at the time of the pregnancy seems not to impact on the outcome: women who carried to term were not significantly older compared to those having spontaneous abortion, (mean age 26.2 ± 6.3 vs 24.1 ± 2.1, *p* = 0.609), miscarriage (mean age 26.2 ± 6.3 vs 23.8 ± 5.8, *p* = 0.647) or VPT (mean age 26.2 ± 6.3 vs 24.3 ± 5.5, *p* = 0.553).

The marital status seems to impact on the pregnancy outcome: in fact, 71.2% of women who carried to term were married (*p* < 0.001) and 63.5% of them reported a previous pregnancy (*p* = 0.006). Conversely, women who decided to undergo self-induced abortion or VPT were unmarried in 76.5% (*p* < 0.001) and 65.4% (*p* = 0.027) of cases, respectively. Notably, also women who reported a spontaneous abortion were unmarried in the majority of cases (66.7%). Accordingly, 74% of Eritrean women (who were more likely to carry the pregnancy to term) resulted to be married (vs 25% of Nigerian women, *p* < 0.001) and 13 of them (42%) already had a child, compared to 16/79 (20%) women from other countries (*p* = 0.018). Only 17.6% of women who practiced a self-induced abortion had already a child (*p* < 0.001).

Another interesting point raised from our analysis was the role of educational status on pregnancy outcome. Low education attainment was not associated with a higher probability of unsafe abortion, when compared to the educational levels of those who performed medical abortions (*p* = 0.447). Nonetheless, Eritrean women, who were more likely to carry the pregnancy to term, attended school with a mean of 8 ± 4.6 years, compared to Nigerian women and women from other Countries who reported 6 ± 5.1 and 5 ± 4.1 years of education (*p* = 0.046 and *p* = 0.001, respectively). Finally, 25 Eritrean women (81%) were pregnant on arrival at ASC and ERCs whereas Nigerian women (47; 77%) got pregnant during their stay (*p* < 0.001).

### Delivery Outcome

According to the 2010 European Perinatal Health Report (Euro-PERISTAT), indicators of delivery outcome were reported in Table 3 [20, 21]. No significant differences were observed in term of different nationalities, ages and other demographic characteristics.

**Table 3:** Euro-PERISTAT indicators (Modified from [20, 21]

INDICATORS	
Foetal, neonatal and child health	N (%)
Foetal mortality	0 (0%)
Neonatal mortality	0 (0%)
Newborn infants with low birth weight (< 2500 g)	8 (15.4%)
Somatic gestational age (< 37 weeks)	4 (7.7%)
Prevalence of selected congenital anomalies	0 (0%)
5th minutes Apgar scores (< 7)	0 (0%)
Foetal and neonatal deaths due to congenital anomalies	0 (0%)
Prevalence of cerebral palsy	0 (0%)
Maternal health	
Maternal mortality	0 (0%)
Incidence of severe maternal morbidity	0 (0%)
Population characteristics/risk factors	
Multiple birth rate by number of foetuses	0 (0%)
Distribution of maternal age:	
*15–17*	1 (1.9%)
*18–19*	1 (1.9%)
20–24	23 (44.2%)
25–29	16 (30.8%)
30–34	6 (11.5%)
35–39	2 (3.9%)
40–44	3 (5.8%)
45+	0 (0%)
Distribution of parity:	
0	23 (44.2%)
1–4	26 (50%)
> 5	3 (5.8%)
Percentage of women who smoked during pregnancy	2 (3.9%)
Mothers’ educational level; years (median, IQR)	7 (0–13)
Distribution of mothers’ country of birth:	
Nigerian	19 (36.5%)
Eritrean	24 (46.2%)
Others	9 (17.3%)
Mothers’ prepregnancy body mass index:	
Underweight <18.50	8 (15.3%)
Normal range 18.50–24.99	37 (71.2%)
Overweight 25.00–29.99	4 (7.7%)
Obese >30.00	3 (5.8%)
Health care services2	
Mode of delivery:	
Vaginal, non instrumental	44 (84.6%)
Vaginal: forceps	0 (0%)
Vaginal: ventouse	1 (1.9%)
Caesarean: before or at onset of labour/elective	5 (9.6%)
Caesarean: during labour/emergency	2 (3.9%)
Infants breast fed at birth (exclusively breastfed)	46 (88.5%)

## Discussion

Physiological or pathological pregnancies are among the main reasons for seeking medical care in migrant women recently arrived in the host countries, thus significantly engaging the host national health systems. Moreover, their outcomes appear to be largely influenced by the public health policies adopted [20]. For this reason, detailed information on the topic appears to be crucial for optimal management of pregnancies in the migrant population. Our data are in line with the percentage of migrants arriving in Italy, [1], in the terms of the women’s origins: Nigeria (61; 55.5%) vs Eritrea (31; 28.2%).

This is the first study from Italy’s reception Centers quantifying differences in pregnancy outcomes between newly arrived migrant women of different origin. Among ethnic groups, data showed that Nigerian women were at a significantly higher risk for abortive outcomes: VPT, miscarriage and self-induced abortion. The aetiological factors for this observation are complex. International Organization for Migration (IOM) estimates that about 80% of Nigerian women who arrived by sea in 2016 were likely to be victims of trafficking for sexual exploitation in Italy or in other countries of the European Union. In addition, an increased number of sexual violence cases by persons outside the trafficking network were reported in Libya involving women and minors. It is undeniably true that these conditions expose them to a high rate of unwanted pregnancies [22]. Usually, women continue to follow the same preventive and reproductive patterns as found in their countries of origin. Likewise, many of the migrant women who wished to terminate an unintended pregnancy may have a direct or an indirect experience of previous self-induced abortion in their own countries [23]. According to The Society of Gynaecologists and Obstetricians of Nigeria 610,000 unsafe abortions a year are carried out in Nigeria, and the death rate is thought to be one of the highest in Africa [24].

Furthermore, the group of women undergoing self-induced abortion or VPT were mostly unmarried. This data could suggest that unmarried women could represent a particularly socially fragile population among migrants, who could be also reluctant to report a self-induced abortion (misclassified as spontaneous). On the contrary, migrant women who were married or with already at least one child, had higher delivery rates. There are some possible explanations. A steady family background may represent a relevant protective factor in Eritrean women who were more likely to carry the pregnancy to the term. In some countries, having children is the ultimate goal of marriage and symbolizes femininity [25]. Collaterally, it should be noted that given particular personal situations i.e. pregnant women, single parents with minor children as in other vulnerable conditions, an access to a range of benefits in reception procedure could be facilitated.

It is well known that the level of literacy among migrant populations is directly or indirectly related to health outcomes. Low levels of health literacy can influence access to reproductive health services even when information is presented to women in their language of origin [26]. In contrast to the statement above, the study of Ajayi et al. reveals that middle-class young women, specifically university students, were prone to unplanned pregnancy, use of unsafe emergency contraception methods and unsafe abortion [27]. It is also recognized that sexual behaviour is affected by the prevailing social rules of the country of origin, and these rules act ambivalently. Moreover, religion continues to have a strong influence on sexual beliefs and customs [25]. In our population the women who chose to undergo unsafe abortion did not result to have significantly lower educational levels, compared to women who preferred VPT. Different traditional and cultural concepts of pregnancy may explain the different approach to abortion independently of educational level.

The unmarried Nigerian women and unaccompanied girls, are among the most at risk of being victims of traffic for sexual exploitation, although it cannot be excluded that migrants from other nationalities are also affected by trafficking. These circumstance expose them to unintended pregnancies during their stay in reception facilities (or just before, along the way or in Libyan detention centers, when they often are subject to sexual violence) and thus more likely to terminate (legally or not) the pregnancy [22, 28]. Interestingly, the practice of self-practicing abortion is clearly more prevalent among the Nigerian population and is not the result of the education level.

In this report we have not explored women’s motivation in preferring self-induced abortion instead of VPT. Different cultural beliefs and obstacles to accessing VPT (beta -HCG confirmation, ultrasound to determine gestational age and paranaesthesia visit) could be the real cause rather than the lack of knowledge about Italian abortion laws (*legge 104/1978*).

Nigeria contributes 14% of the total maternal deaths in the world [29], 21.3% of which are attributed to septic abortion [30]. The practice of self-induced abortion is very frequent probably due to restrictive abortion law in terms of medical abortion [31], low level of awareness and knowledge of contraception and underutilisation of the emergency one [27, 32], due to moral-religious stigma involved in the purchase of contraceptives and condoms. In addition the belief that certain substances such as “concoction” (a mixture of substances with unproven efficacy, such as salt and hot water, soft drinks, a local brand of analgesic known as Alabukun, lime and potash, and lime and Alabukun) can be effective as a contraceptive, leads Nigerian women to have a low control of their fertility and therefore often opting for voluntary termination of pregnancy [27].

In Italy, the most common method among Nigerian women for self-induced abortion is the oral intake or vaginal self-application of prostaglandins used for the treatment of gastric pyrosis [14, 33] purchased on the black market online or through traffickers. The idea that self-induced abortion could be easier, faster and non-invasive with less impact on daily routine (absence from the street for the days required for intervention and convalescence, not allowed by the trafficking network) [22] may represent one of the causes of the high percentage of self-induced abortions in some settings of asylum seekers in Italy.

Our study demonstrates the need to take action in order to support migrant women’s reproductive health especially in family planning and abortion services. In ASC and ERCs the Medical service is available *24 h*, seven days a week, but we acknowledge that access to information, prevention and treatment services must be improved. Health care professionals need more information on how to better take into account migrant women’s special needs such as information on family planning and reliable contraceptive methods, considering different religious and cultural customs of arriving migrants. Moreover, enhancement of psychosocial services could be the way forward to improve outcomes in reproductive health.

Our study has some important limitations that need to be considered in interpreting the results. The major limitation of this study is the small size of the population, which may be responsible for low statistical power thereby weakening our conclusions. Unfortunately, we were unable to follow-up all pregnancy outcomes, as 50.5% of pregnant women dropped-out from the study because they moved before the pregnancy outcome was known. The asylum-seeker population is subjected to frequent relocation and even tracking via personal mobile phone has proved unsuccessful, as the mobile number is often changed. The data we obtained represented only a very small percentage of the pregnant women who were lost to follow up, therefore they were not taken into account. Migrants transferred abroad according to European relocation system were not longer traceable. Nevertheless, the baseline characteristic of pregnant women lost to follow up did not differ from the study group. The authors cannot exclude information bias on self-induced/spontaneous abortions due to self-reporting. Hence, the rate of self-induced may have been under-reported among the participants. Although the study was conducted in five large reception centers in Italy, its conclusions may not be directly extensible to all asylum seekers present in Italy and Europe, being potentially influenced not only by the reception modalities and the health facilities available, but also and above all by the anthropological and cultural specificities of the people hosted.

To the best of our knowledge, this is one of the few population-based studies on pregnancy outcomes in newly arrived migrants in Europe. The findings may not be directly generalized to other settings but provide precious information about the particularly socially fragile population among migrants.

## Conclusions

The progressive increase in migratory flows and the growing demand for health from migrants creates additional pressure on the health facilities of the host countries. The asylum-seeker population is a dynamic group subjected to frequent relocation around the host country or abroad. The findings from this study should alert health professionals looking after Nigerian women, young and unmarried, with symptoms of pregnancy or with ongoing abortion, as they might be at high risk of infection and maternal mortality. The lack or failure of educational pathways for migrant women can lead the health system to face unexpected problems and at the cost of human lives and resources greater than anticipated. The drafting of policies aimed at rationalizing the provision of health services seems to be the best possible response for managing the additional workload. Accurate knowledge of the health status of migrants is the key prerequisite for the creation of such policies and for the implementation of health promotion programs aimed at reducing the knowledge gap between resident and migrant population in terms of health rights. The awareness of the variables that may affect pregnancy outcomes is critical to identify entry points for interventions for maternal health in migrants in the WHO European Region. Further insights should be obtained into what additional programmes should be developed, to address the reproductive health needs of migrant groups.
